# Intra-axial primary brain tumor differentiation: comparing large language models on structured MRI reports vs. radiologists on images

**DOI:** 10.1007/s00330-025-11924-3

**Published:** 2025-08-22

**Authors:** Takeshi Nakaura, Hiroyuki Uetani, Naofumi Yoshida, Naoki Kobayashi, Yasunori Nagayama, Masafumi Kidoh, Jun-Ichiro Kuroda, Akitake Mukasa, Toshinori Hirai

**Affiliations:** 1 Department of Diagnostic Radiology, Graduate School of Medical Sciences, Kumamoto University, Kumamoto, Japan; 2 Department of Neurosurgery, Graduate School of Medical Sciences, Kumamoto University, Kumamoto, Japan

**Keywords:** Artificial intelligence, Large language models, Brain tumors, Structured reports, Magnetic resonance imaging

## Abstract

**Objective:**

Aimed to evaluate the potential of large language models (LLMs) in differentiating intra-axial primary brain tumors using structured magnetic resonance imaging (MRI) reports and compare their performance with radiologists.

**Materials and methods:**

Structured reports of preoperative MRI findings from 137 surgically confirmed intra-axial primary brain tumors, including Glioblastoma (*n* = 77), Central Nervous System (CNS) Lymphoma (*n* = 22), Astrocytoma (*n* = 9), Oligodendroglioma (*n* = 9), and others (*n* = 20), were analyzed by multiple LLMs, including GPT-4, Claude-3-Opus, Claude-3-Sonnet, GPT-3.5, Llama-2-70B, Qwen1.5-72B, and Gemini-Pro-1.0. The models provided the top 5 differential diagnoses based on the preoperative MRI findings, and their top 1, 3, and 5 accuracies were compared with board-certified neuroradiologists’ interpretations of the actual preoperative MRI images.

**Results:**

Radiologists achieved top 1, 3, and 5 accuracies of 85.4%, 94.9%, and 94.9%, respectively. Among the LLMs, GPT-4 performed best with top 1, 3, and 5 accuracies of 65.7%, 84.7%, and 90.5%, respectively. Notably, GPT-4’s top 3 accuracy of 84.7% approached the radiologists’ top 1 accuracy of 85.4%. Other LLMs showed varying performance levels, with average accuracies ranging from 62.3% to 75.9%. LLMs demonstrated high accuracy for Glioblastoma but struggled with CNS Lymphoma and other less common tumors, particularly in top 1 accuracy.

**Conclusion:**

LLMs show promise as assistive tools for differentiating intra-axial primary brain tumors using structured MRI reports. However, a significant gap remains between their performance and that of board-certified neuroradiologists interpreting actual images. The choice of LLM and tumor type significantly influences the results.

**Key Points:**

***Question*** *How do Large Language Models (LLM) perform when differentiating complex intra-axial primary brain tumors from structured MRI reports compared to radiologists interpreting images?*

***Findings**** Radiologists outperformed all tested LLMs in diagnostic accuracy. The best model, GPT-4, showed promise but lagged considerably behind radiologists, particularly for less common tumors*.

***Clinical relevance**** LLMs show potential as assistive tools for generating differential diagnoses from structured MRI reports, particularly for non-specialists, but they cannot currently replace the nuanced diagnostic expertise of a board-certified radiologist interpreting the primary image data*.

**Graphical Abstract:**

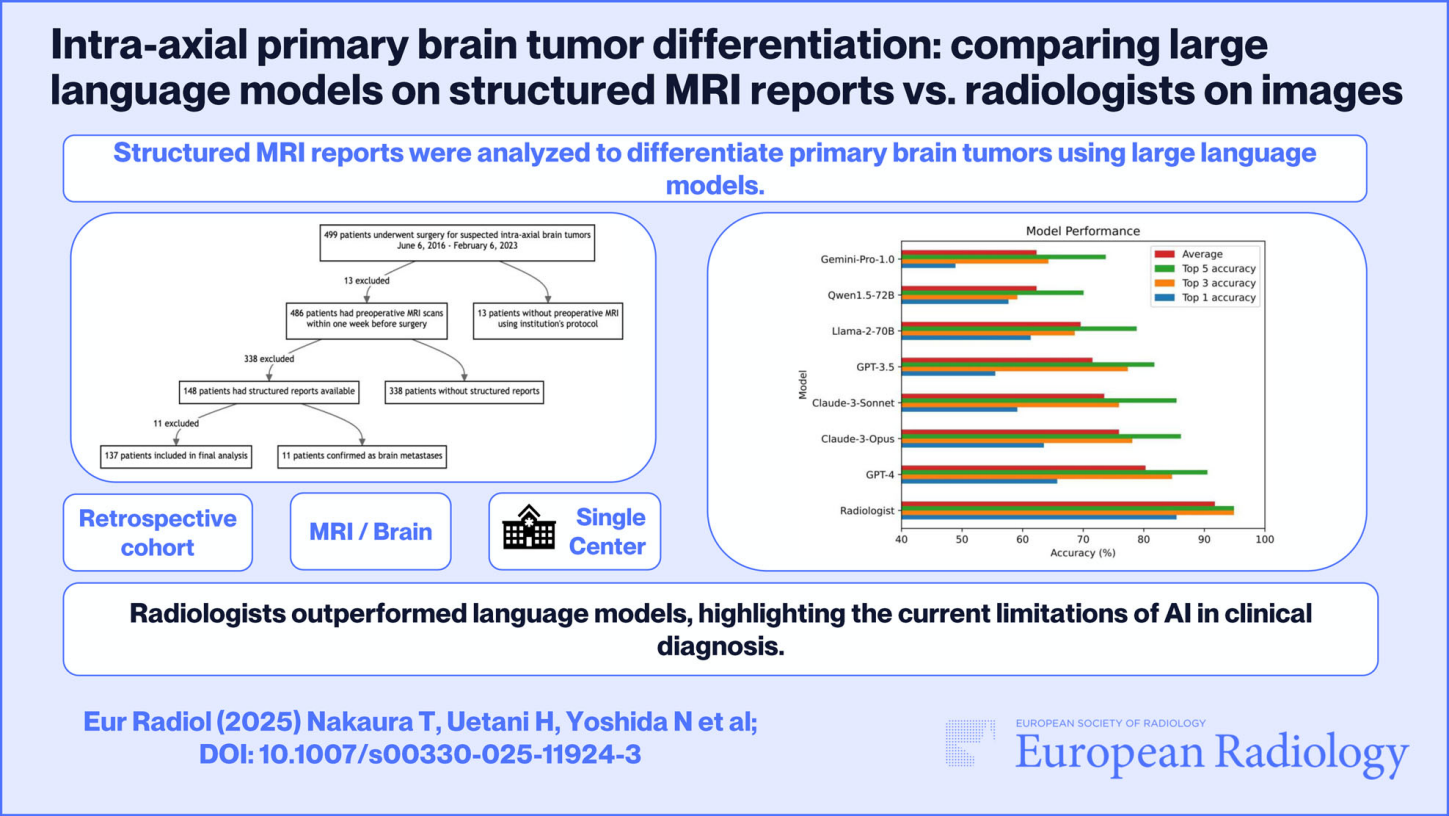

## Introduction

The accurate diagnosis of intra-axial primary brain tumors is a critical yet challenging task in neuroradiology [1, 2]. These tumors, which originate within the brain parenchyma, exhibit a wide range of imaging characteristics that can often overlap, making definitive diagnosis difficult. MRI plays a pivotal role in the evaluation of these lesions, providing detailed information about tumor morphology, composition, and surrounding tissue involvement [3].

Recent advancements in artificial intelligence, particularly large language models (LLMs), have shown promise in various medical applications [4–6]. These models, trained on vast amounts of textual data, have demonstrated impressive capabilities in natural language understanding and generation. However, current multimodal LLMs, which can process both text and images, still have limited diagnostic capabilities when it comes to direct interpretation of medical images [7, 8]. Given these limitations, structured MRI reports offer a potential alternative for AI-assisted diagnosis. These reports, which contain standardized descriptions of imaging findings, could serve as a proxy for actual image interpretation. A study on structured reporting in neuroradiology demonstrated that this approach ensures reliable detection and documentation of brain pathologies, highlighting its clinical feasibility [9]. Recently, one study has explored the utility of LLMs in providing differential diagnoses based on radiologists’ MRI reports for various common brain tumors, demonstrating promising results [10]. However, this investigation has primarily focused on a broad range of conditions or more common pathologies that are generally easier to differentiate. Furthermore, guidelines for primary brain tumors are frequently updated [11, 12], raising questions about the ability of LLMs to adapt to these evolving diagnostic criteria and classification systems. To date, there has been a lack of studies specifically examining the performance of LLMs in differentiating intra-axial primary brain tumors, which are known for their clinical complexity and diagnostic challenges. Additionally, numerous LLMs, including open-source models, have been recently released to the public [13–16]. However, their respective capabilities in interpreting structured reports, particularly in the context of brain tumor diagnosis, have not been thoroughly evaluated. This gap in knowledge presents an important area for investigation, as understanding the strengths and limitations of various LLMs could inform their potential integration into clinical workflows.

This study aimed to evaluate the potential of LLMs as assistive tools in differentiating intra-axial primary brain tumors using structured MRI reports.

## Materials and methods

### Study design and patient

**Fig. 1:**
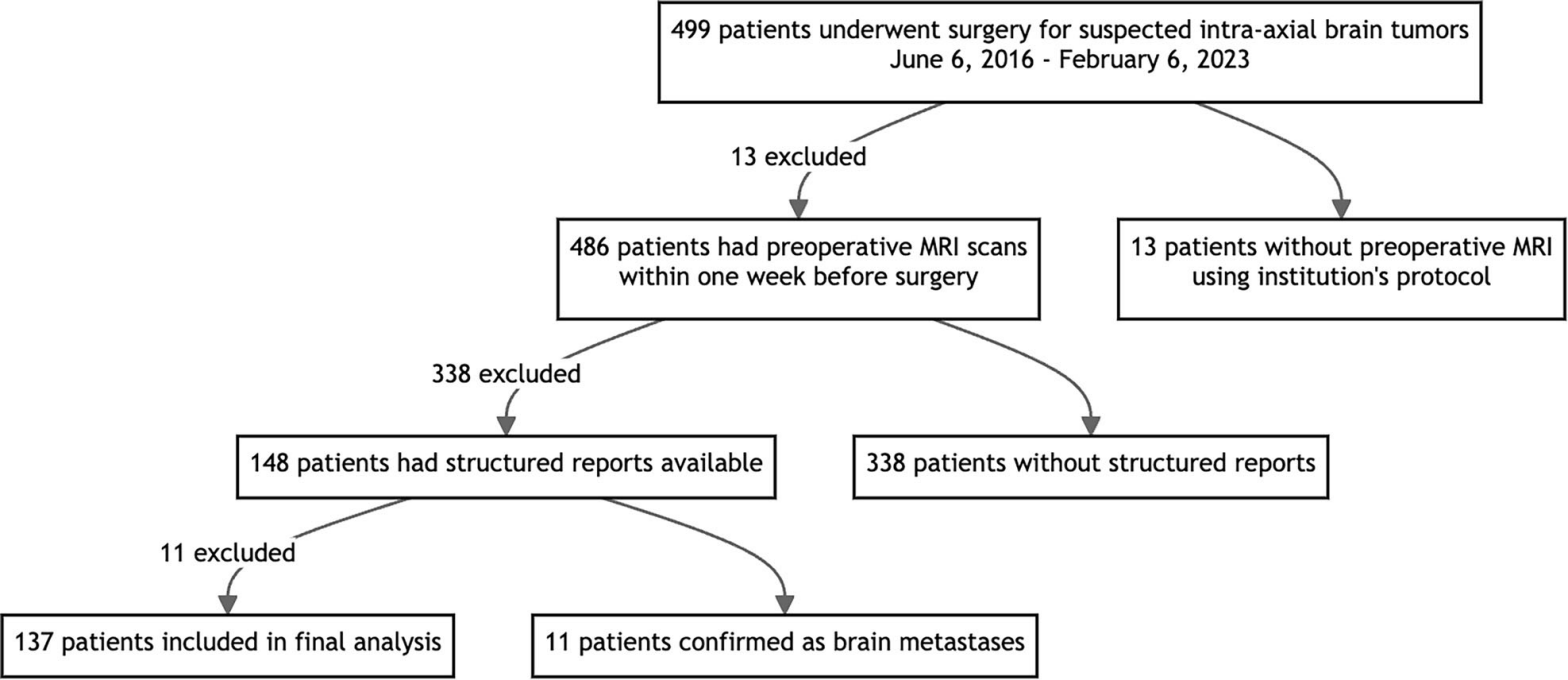
Patient selection workflow. This flowchart illustrates the process of patient selection for the study of intra-axial primary brain tumors. From an initial pool of 499 patients who underwent surgery for suspected intra-axial brain tumors between June 6, 2016, and February 6, 2023, the study narrowed down to a final cohort of 137 patients. The selection process involved several steps: ensuring preoperative MRI scans were performed within one week before surgery using the institution’s primary brain tumor evaluation protocol (486 patients), identifying cases with available structured reports (148 patients), and excluding cases postoperatively confirmed as brain metastases (11 cases excluded). This rigorous selection process ensured that the final study cohort consisted of surgically confirmed intra-axial primary brain tumors with complete and relevant imaging data

This retrospective study was approved by our institutional review board. Informed consent for this retrospective study was waived by our institutional ethics committee. This retrospective study focused on surgically confirmed intra-axial primary brain tumors. From June 6, 2016, to February 6, 2023, 499 patients underwent surgery on the suspicion of intra-axial brain tumors. Of these, 486 patients had preoperative MRI scans using our institution’s primary brain tumor evaluation protocol within one week before surgery. Among these 486 cases, 148 had structured reports available. After excluding 11 cases that were postoperatively confirmed as brain metastases, a final cohort of 137 patients was included in the analysis (Fig. 1).

### MRI protocol

Brain MRI scans were performed using either a 3.0-T Ingenia scanner (Philips Healthcare) or a 3.0-T MAGNETOM Prisma scanner (Siemens Healthineers) with our institution’s primary brain tumor evaluation protocol. This imaging protocol included, at minimum, the following sequences: 2D T1-weighted imaging, T2-weighted imaging, fluid-attenuated inversion recovery, diffusion-weighted imaging, gadolinium-enhanced T1-weighted imaging, 3D gadolinium-enhanced T1-weighted imaging, magnetic resonance spectroscopy, and dynamic susceptibility contrast perfusion imaging. Additional sequences, such as diffusion tensor imaging, were performed as needed based on clinical indications and the radiologist’s discretion. Each structured report included the tumor’s anatomical location, size measurements, signal intensities on T1-weighted, T2-weighted, and FLAIR sequences, contrast enhancement patterns, and spectroscopy data. An anonymized example of a structured report is provided in the Supplementary Material.

### Data input to LLM

**Fig. 2:**
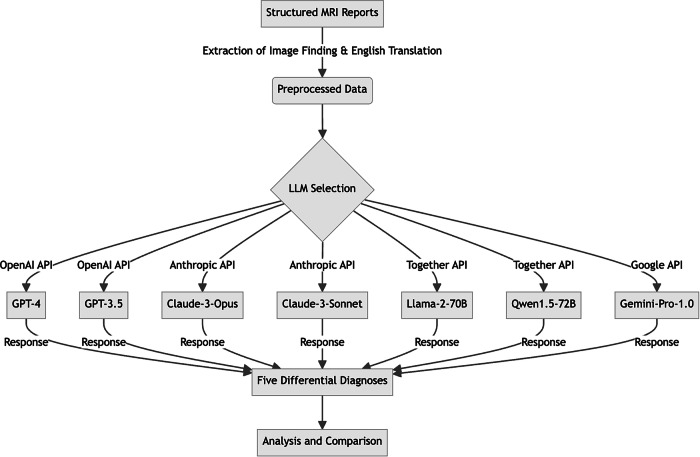
Workflow diagram of LLM API calls for differential diagnosis generation. This flowchart illustrates the process of utilizing various Large Language Models (LLMs) to generate differential diagnoses from structured MRI reports. The workflow begins with the extraction of image findings from structured MRI reports and their translation into English. This preprocessed data is then fed into multiple LLMs through different API calls: GPT-4 and GPT-3.5 via OpenAI API, Claude-3-Opus and Claude-3-Sonnet via Anthropic API, Llama-2-70B and Qwen1.5-72B via Together API, and Gemini-Pro-1.0 via Google API. Each LLM processes the input and generates a response containing five differential diagnoses. These responses are then collected for further analysis and comparison. This approach allows for a comprehensive evaluation of different LLMs’ capabilities in interpreting medical imaging reports and generating potential diagnoses

The structured reports used in this study were originally created by a board-certified neuroradiologist with 16 years of experience in brain MRI diagnostics. The image findings section of the preoperative MRI structured reports was extracted to input into multiple LLMs. The structured reports, originally drafted in Japanese, were translated into English by a radiologist with over 20 years of experience in writing English manuscripts. Ambiguous portions were subsequently verified by a neuroradiologist with more than 10 years of experience in English-language research to ensure accuracy and consistency in the input data. The following LLMs were utilized in this study, representing the most prominent closed and open-source LLMs available as of March 2024. We used Python libraries “*langchain*” (version 0.0.100) to access the Application Programming Interfaces (API) on the websites of OpenAI (https://openai.com), Google (https://ai.google.dev), Anthropic (https://www.anthropic.com), and Together (https://www.together.ai), calling multiple models. These libraries and API calls allowed us to seamlessly integrate and compare the performance of these diverse language models in our study (Fig. 2). Each model was provided with the structured report data and asked to generate a list of five potential diagnoses, ranked by likelihood. The models range from OpenAI’s Generative Pre-trained Transformer (GPT) series [13, 17, 18] to Anthropic’s Claude models [14], Meta’s open-source Llama-2 [15], Alibaba’s Qwen [16], and Google’s Gemini [19]. Table 1 shows details of these models. We used the following prompt for LLMs: “As a radiologist, you are required to provide a concise and prioritized catalog of differential diagnoses without a corresponding disease description, comprising no more than five suspects, ranked by level of suspicion.” This prompt was selected to ensure clarity and uniformity. To manage the reproducibility inherent in LLM responses, we set the temperature of all LLMs to ‘zero’ [20, 21]. For this study, all LLMs were accessed via their respective APIs and used in their default configurations without any additional task-specific fine-tuning.

**Table 1:** Model specifications and access information

Model name	Accessed site	Model name on site	Knowledge cutoff
GPT-3.5	OpenAI (https://openai.com)	gpt-3.5-turbo-0125	Apr 2023
GPT-4	OpenAI (https://openai.com)	gpt-4-0125-preview	Dec 2023
Claude-3-Opus	Anthropic (https://www.anthropic.com)	claude-3-opus-20240229	Aug 2023
Claude-3-Sonnet	Anthropic (https://www.anthropic.com)	claude-3-sonnet-20240229	Aug 2023
Llama-2-70B	Together (https://www.together.ai)	togethercomputer/llama-2-70b-chat	Jul 2023
Qwen1.5-72B	Together (https://www.together.ai)	Qwen/Qwen1.5-72B-Chat	Apr 2023
Gemini-Pro-1.0	Google (https://ai.google.dev)	gemini-pro	Nov 2023

### Data analysis

The differential diagnoses generated by the LLMs and those provided by radiologists were compared against the final postoperative diagnoses to evaluate their accuracy. We calculated the Top 1, Top 3, and Top 5 accuracy rates for each LLM and the radiologists. The top-1 accuracy was defined as the percentage of cases in which the first suggested diagnosis by the LLM or radiologist matched the final postoperative diagnosis. Similarly, the top-3 and top-5 accuracies represent the percentages of cases where the correct diagnosis was present among the top three or five suggestions, respectively. The Top 1 accuracy represents the percentage of cases where the correct diagnosis was listed as the most likely option. Top 3 and Top 5 accuracies indicate the percentage of cases where the correct diagnosis appeared within the top three or five suggestions, respectively. Additionally, we also calculated average accuracy (average of Top 1, Top 3, and Top 5 accuracies) to evaluate the mean performance of all models. We also computed these accuracy metrics for three subcategories of brain tumors: Glioblastoma, CNS Lymphoma, and Other Primary Brain Tumors. These analyses were performed to assess the performance of each LLM and compare it to that of radiologists across different types of brain tumors.

The cases in our dataset, spanning from June 6, 2016, to February 6, 2023, included a mix of WHO 2016 and 2021 classification terminologies. Additionally, the LLMs’ differential diagnoses often used older classifications. We converted these to the newer classification system, where possible, with the WHO 2021 classification system when feasible. For example, cases previously classified as Anaplastic Astrocytoma were reclassified as Astrocytoma, IDH-mutant, CNS WHO grade 3 in the new system. Similarly, Oligodendroglioma cases were updated to Oligodendroglioma, IDH-mutant and 1p/19q-codeleted, with CNS WHO grade 2 or 3. Regarding Glioblastoma, we noted that the LLMs rarely specified IDH wildtype or mutant status in their outputs. Additionally, some pathological reports included Glioblastoma IDH-mutant classifications. In the WHO 2021 classification system, these might correspond to Astrocytoma, IDH-mutant, grade 4. However, for our analysis, we maintained the Glioblastoma IDH-mutant classification to align with the available pathological data.

Additionally, to evaluate the alignment of each model with the WHO 2021 classification system, we examined cases where the final diagnosis was either Glioblastoma or Astrocytoma. We specifically focused on whether the outputs of LLMs included ‘IDH’ status, which is crucial for distinguishing between IDH wildtype and IDH mutant tumors. This analysis aimed to identify any discrepancies in the models’ classifications and ensure that they accurately reflected the updated diagnostic criteria.

## Results

A total of 137 patients (70 males, 67 females) with a mean age of 58.7 ± 17.2 years were included in this study. The final diagnoses of the intra-axial primary brain tumors varied widely, with Glioblastoma (IDH-wildtype or IDH-mutant) being the most common (*n* = 77), followed by CNS Lymphoma (*n* = 22). Other diagnoses included Astrocytoma (IDH-mutant, CNS WHO grade 3: *n* = 8; grade 2: *n* = 1), Oligodendroglioma (IDH-mutant and 1p/19q-codeleted, CNS WHO grade 3: *n* = 4; grade 2: *n* = 5), Hemangioblastoma (*n* = 6), and Diffuse Midline Glioma, H3 K27-altered (*n* = 4). Less common tumors included Ependymoma (*n* = 3), Ganglioglioma (*n* = 2), and single cases of various other tumor types such as Diffuse Hemispheric Glioma, H3 G34-mutant, Central Neurocytoma, and Pilocytic Astrocytoma. The detailed patient characteristics and final diagnoses are presented in Table 2.

**Table 2:** Patient characteristics and final diagnoses

Characteristic	Value
Age, years (mean ± SD)	58.7 ± 17.2
Sex, *n*	
Male	70
Female	67
Final diagnoses, *n*	
Glioblastoma, IDH-wildtype or IDH-mutant	77
CNS lymphoma	22
Astrocytoma, IDH-mutant, CNS WHO grade 3	8
Astrocytoma, IDH-mutant, CNS WHO grade 2	1
Oligodendroglioma, IDH-mutant and 1p/19q-codeleted, CNS WHO grade 3	4
Oligodendroglioma, IDH-mutant and 1p/19q-codeleted, CNS WHO grade 2	5
Hemangioblastoma	6
Diffuse midline glioma, H3 K27-altered	4
Ependymoma	3
Ganglioglioma	2
Diffuse hemispheric glioma, H3 G34-mutant	1
Central neurocytoma	1
Astrocytoma, IDH-wildtype, CNS WHO grade 3	1
Pilocytic astrocytoma	1
CNS tumor with BCOR internal tandem duplication	1

*SD* standard deviation, *CNS* central nervous system, *IDH* isocitrate dehydrogenase, *BCOR* BCL6 corepressor

**Fig. 3:**
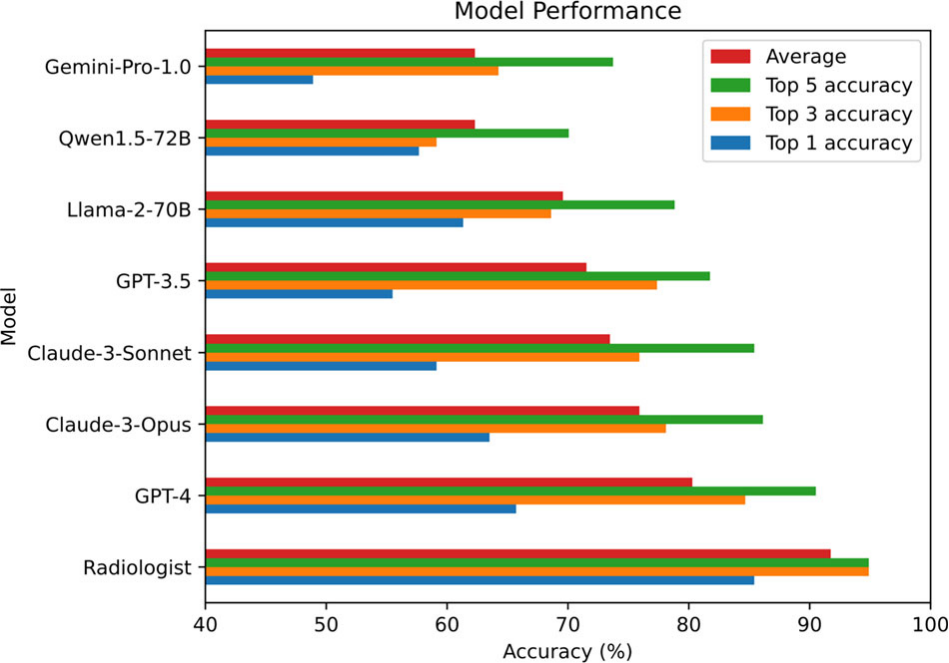
Comparison of overall diagnostic performance between radiologists and various large language models (LLMs) for intra-axial primary brain tumors. The bars represent the top 1, top 3, and top 5 accuracies, as well as the average accuracy across all cases. Radiologists achieved the highest accuracies, with GPT-4 emerging as the top-performing LLM, closely approaching radiologists’ top 1 accuracy with its top 3 accuracy of 84.7%

Table 3 and Fig. 3 present a comprehensive comparison of diagnostic performance between radiologists and various LLMs. Radiologists demonstrated superior performance, achieving top 1, 3, and 5 accuracies of 85.4%, 94.9%, and 94.9%, respectively, with an impressive average accuracy of 91.7%. Among the LLMs evaluated, GPT-4 emerged as the top performer, with top 1, 3, and 5 accuracies of 65.7%, 84.7%, and 90.5%, respectively, resulting in an average accuracy of 80.3%. It is noteworthy that GPT-4’s top 3 accuracy of 84.7% closely approached the radiologists’ top 1 accuracy of 85.4%, highlighting the potential of advanced LLMs in medical diagnosis.

**Table 3:** Model performance comparison

Model	Top 1 accuracy	Top 3 accuracy	Top 5 accuracy	Average
Radiologist	85.4% (90.9%, 95.5%, 68.4%)	94.9% (97.4%, 100.0%, 86.8%)	94.9% (97.4%, 100.0%, 86.8%)	91.7% (95.2%, 98.5%, 80.7%)
GPT-4	65.7% (98.7%, 0.0%, 36.8%)	84.7% (98.7%, 90.9%, 52.6%)	90.5% (98.7%, 100.0%, 68.4%)	80.3% (98.7%, 63.6%, 52.6%)
Claude-3-Opus	63.5% (97.4%, 4.5%, 28.9%)	78.1% (97.4%, 54.5%, 52.6%)	86.1% (97.4%, 100.0%, 55.3%)	75.9% (97.4%, 53.0%, 45.6%)
Claude-3-Sonnet	59.1% (98.7%, 0.0%, 13.2%)	75.9% (98.7%, 50.0%, 44.7%)	85.4% (98.7%, 95.5%, 52.6%)	73.5% (98.7%, 48.5%, 36.8%)
GPT-3.5	55.5% (97.4%, 0.0%, 2.6%)	77.4% (98.7%, 90.9%, 26.3%)	81.8% (100.0%, 100.0%, 34.2%)	71.5% (98.7%, 63.6%, 21.1%)
Llama-2-70B	61.3% (100.0%, 0.0%, 18.4%)	68.6% (100.0%, 45.5%, 18.4%)	78.8% (100.0%, 90.9%, 28.9%)	69.6% (100.0%, 45.5%, 21.9%)
Gemini-Pro-1.0	48.9% (77.9%, 9.1%, 13.2%)	64.2% (83.1%, 50.0%, 34.2%)	73.7% (85.7%, 81.8%, 44.7%)	62.3% (82.3%, 47.0%, 30.7%)
Qwen1.5-72B	57.7% (97.4%, 0.0%, 10.5%)	59.1% (97.4%, 0.0%, 15.8%)	70.1% (97.4%, 45.5%, 28.9%)	62.3% (97.4%, 15.2%, 18.4%)

Values are presented as Total% (Glioblastoma%, CNS lymphoma%, Other primary brain tumors%)

*CNS* central nervous system

**Fig. 4:**
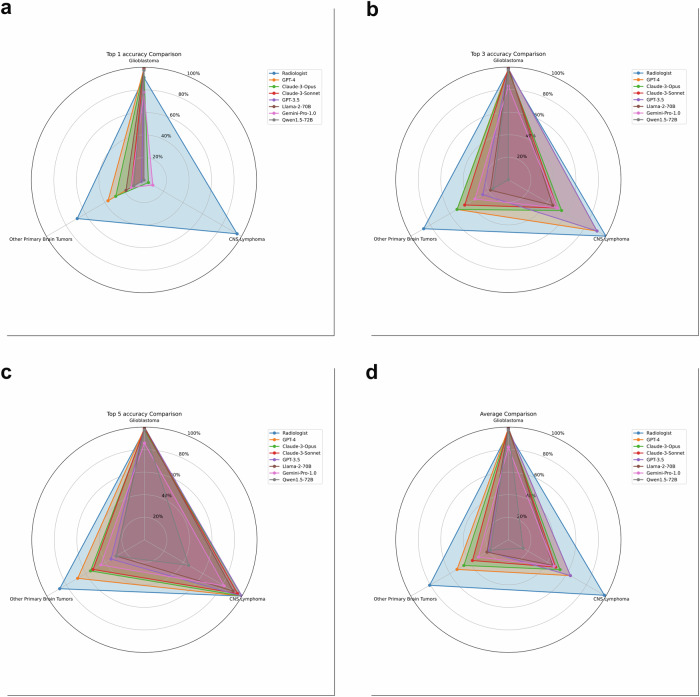
**a** Average accuracy. **b** Top 1 accuracy. **c** Top 3 accuracy. **d** Top 5 accuracy. These graphs illustrate the diagnostic performance of various Large Language Models (LLMs) compared to radiologists across different types of intra-axial primary brain tumors. The average accuracy (**a**) provides an overall view of model performance, while the Top 1 (**b**), Top 3 (**c**), and Top 5 (**d**) accuracies offer insights into the models’ capabilities at different levels of diagnostic precision. The graphs highlight the superior performance of radiologists and the varying capabilities of different LLMs, particularly in distinguishing between Glioblastoma, CNS lymphoma, and other primary brain tumors. They also demonstrate how the accuracy of LLMs improves when considering a broader range of top diagnoses, especially for less common tumor types

However, a closer examination of Table 3 and Fig. 4 reveals a more nuanced picture of the LLMs’ diagnostic capabilities across different tumor types. In the Top 1 accuracy, most LLMs demonstrated high performance primarily for Glioblastoma, with accuracies ranging from 77.9% (Gemini-Pro-1.0) to 100% (Llama-2-70B). In contrast, their ability to correctly identify CNS lymphoma and other primary brain tumors as the top diagnosis was notably lower. For instance, GPT-4, despite having the highest overall Top 1 accuracy among LLMs at 65.7%, achieved 98.7% accuracy for Glioblastoma but 0% for CNS lymphoma and only 36.8% for other primary brain tumors. This pattern was consistent across most LLMs, with many showing 0% Top 1 accuracy for CNS lymphoma. The diagnostic capabilities of LLMs improved when considering the Top 3 accuracies. Here, the models showed better performance in identifying CNS lymphoma, with accuracies ranging from 0% (Qwen1.5-72B) to 90.9% (GPT-4 and GPT-3.5). The accuracy for other primary brain tumors also improved, though more modestly, with ranges from 15.8% (Qwen1.5-72B) to 52.6% (Claude-3-Opus and GPT-4). It was only when considering the Top 5 accuracies that the LLMs demonstrated more balanced performance across all tumor types. For example, GPT-4’s Top 5 accuracy was 98.7% for Glioblastoma, 100% for CNS lymphoma, and 68.4% for other primary brain tumors. Similarly, Claude-3-Opus achieved 97.4%, 100%, and 55.3% for these categories, respectively.

Among the 86 cases with a final diagnosis of Glioblastoma (*n* = 77) or Astrocytoma (*n* = 9), only a few instances of ‘IDH’ status were identified in the outputs generated by the language models. Specifically, ‘IDH’ was included in the outputs for 2 cases by Claude-3-Opus and for 1 case by Gemini-Pro-1.0. This indicates a limited recognition of IDH status in the differential diagnoses provided by the models, highlighting a potential area for improvement in aligning with the WHO 2021 classification standards.

A representative response from LLMs for one Glioblastoma patient is shown in Table 4.

**Table 4:** Representative response from radiologist and LLMs for a glioblastoma patient

Model	Response
Radiologist	Left temporal lobe mass: suspected high-grade glioma (GBM), suspected meningeal dissemination
GPT-4	1. Glioblastoma multiforme (GBM) 2. Metastatic brain tumors 3. Primary central nervous system lymphoma (PCNSL) 4. Anaplastic astrocytoma 5. High-grade glioma (not otherwise specified)
Llama-2-70B	Based on the provided structured report, the differential diagnoses for the brain tumor, ranked by level of suspicion, are: 1. Glioblastoma (high suspicion): The presence of a large tumor in the left temporal lobe, with inhomogeneous signal intensity, necrosis, and prominent blood vessels, is consistent with glioblastoma. The elevated Cho and Lac levels and decreased NAA level on MRS also support this diagnosis. 2. Metastatic carcinoma (moderate suspicion): The presence of multiple enhancing lesions on contrast-enhanced T1-weighted images, with a ring-shaped enhancement effect, suggests metastatic disease. The location of the lesions near the brainstem and in the left parietal lobe sulcus increases the likelihood of metastasis. 3. Lymphoma (moderate suspension): The presence of a large tumor with high signal intensity on T2-weighted and FLAIR images, with diffusion restriction and a high ADC value, is consistent with lymphoma. The elevated Cho level on MRS also supports this diagnosis. 4. Meningioma (low suspicion): The presence of a tumor in the left temporal lobe, with a homogeneous signal intensity on T1-weighted and T2-weighted images, is consistent with meningioma. However, the presence of necrosis and prominent blood vessels argues against this diagnosis. 5. Brain abscess (low suspicion): The presence of a ring-shaped enhancement effect on contrast-enhanced T1-weighted images, with clustered dot-like low signal areas on SWIp, suggests a brain abscess. However, the absence of surrounding edema and the presence of necrosis argue against this diagnosis. It is important to note that this list represents a differential diagnosis, and the final diagnosis can only be determined after further clinical, radiological, and histopathological evaluations.
Claude-3-Opus	1. Glioblastoma multiforme (GBM) 2. Metastatic brain tumor 3. Anaplastic astrocytoma 4. Primary central nervous system lymphoma (PCNSL) 5. Tumefactive demyelinating lesion (TDL)
Claude-3-Sonnet	Based on the provided structured report, the prioritized catalogue of differential diagnoses is as follows: 1. Glioblastoma 2. Metastatic brain tumor 3. Primary brain lymphoma 4. High-grade glioma (e.g., anaplastic astrocytoma) 5. Atypical meningioma
GPT-3.5	1. Glioblastoma multiforme 2. Metastatic brain tumor 3. Lymphoma 4. Brain abscess 5. Radiation necrosis
Gemini-Pro-1.0	1. Metastatic brain tumor 2. Glioblastoma 3. High-grade glioma 4. Anaplastic astrocytoma 5. Anaplastic oligodendroglioma
Qwen1.5-72B	1. Glioblastoma 2. Metastatic brain tumor 3. Brain abscess 4. Meningioma 5. Astrocytoma

## Discussion

This study provides valuable insights into the potential application of LLMs in the differentiation of intra-axial primary brain tumors using the image finding part of the preoperative MRI structured reports. Our findings reveal both the promising aspects and limitations of these advanced LLMs in medical diagnosis.

The rapid advancement of artificial intelligence presents a unique opportunity for its integration into medical diagnostics, including brain tumor MRI analysis [22]. Since the emergence of ChatGPT, LLMs have garnered the most attention in the field of artificial intelligence, with some reports highlighting their utility in interpreting structured reports [23, 24]. However, the complexity of brain tumor diagnoses, compounded by the frequent updates to diagnostic criteria, poses significant challenges. The evolving landscape of neuro-oncology necessitates that any assistive tool, including LLMs, remain current with the latest guidelines and classifications. This dynamic and diagnostically challenging environment appears to be highly suitable for testing the capabilities of LLMs in clinical settings.

Despite these challenges, recent LLMs demonstrate considerable potential as assistive tools for generating differential diagnoses in our study. Their ability to analyze structured MRI reports allows for the identification of key features that may inform diagnostic decisions. In this study, LLMs, particularly GPT-4, showed promising results in distinguishing common tumors like Glioblastoma and achieved high accuracy in identifying the top 3 or 5 differential diagnoses. These results suggest that LLMs could serve as valuable adjuncts, especially for non-neurospecialized radiologists, enhancing the diagnostic process by providing additional insights and supporting the generation of differential diagnoses. This is a similar result to a feasibility study that used simple imaging patterns to present differential diagnoses to LLMs [25]. The integration of LLMs into clinical workflows could streamline the diagnostic process, allowing radiologists to focus on more complex cases while relying on LLMs for preliminary assessments. The notable increase in accuracy from Top-1 to Top-3 and Top-5 suggests that while pinpointing the single most likely diagnosis remains challenging for LLMs based on text reports alone, they show considerable strength in generating a relevant list of differential diagnoses, which could be particularly useful in ruling possibilities in or out. Furthermore, this assistive capability could be particularly valuable for less experienced practitioners, potentially helping to enhance diagnostic support and service quality.

However, the current limitations of LLMs in performing diagnostic tasks independently are evident. The stark contrast in Top 1 diagnostic accuracy for brain tumors, particularly for less common types such as CNS lymphoma, highlights the challenges LLMs face in achieving the level of expertise exhibited by board-certified radiologists. These limitations may stem from the probabilistic nature of LLM training… As a result, while LLMs can assist in generating differential diagnoses, they are less likely to prioritize rare diseases as the top diagnosis. It is also plausible that the observed performance difference between common tumors like Glioblastoma and less common ones like CNS lymphoma reflects biases present in the models’ extensive training data, which likely mirror real-world incidence rates, potentially leading to better performance on more frequently encountered pathologies. The limited recognition of IDH status in the differential diagnoses provided by the models is a notable finding. The inability of LLMs to adapt to updated guidelines further complicates their role as standalone diagnostic tools. While we cannot fully explain these phenomena, several potential reasons may exist. These limitations may stem from the probabilistic nature of LLM training, which involves unsupervised learning where parts of the input data are masked and filled with high-probability tokens [17, 26]. This approach does not inherently account for temporal relationships or the nuances of clinical practice. As a result, while LLMs can assist in generating differential diagnoses, they are less likely to prioritize rare diseases as the top diagnosis. Furthermore, the vast training data likely includes a significant amount of medical literature based on outdated guidelines, and it is challenging for LLMs to learn the relationships between these and more recent guideline-based texts [4, 27, 28]. Interestingly, given the knowledge cutoff dates of the models used in this study, the WHO 2021 classification should have been included in their training data. However, the models’ performance suggests that mere inclusion of updated information does not guarantee its effective application. To address these issues, specialized LLMs fine-tuned with the latest medical knowledge may be necessary, ideally incorporating a system that indicates which diagnostic criteria the model’s recommendations are based on. Such a system could potentially alleviate concerns about the interpretability of LLM outputs [4], as it would enable clinicians to understand the rationale behind the model’s recommendations. Addressing these limitations will be crucial for the successful integration of LLMs into medical diagnostics, ensuring that they complement rather than replace the critical role of human expertise in patient care.

Recent reviews on AI in medical imaging have highlighted both the potential and the current limitations of LLMs in diagnostic applications [4, 27]. Our findings—demonstrating promising accuracy for common tumors such as glioblastoma but lower performance for less common tumors—align with these reviews. In addition, emerging multimodal LLMs, such as Med-Palm 2 [29], which is specifically designed for healthcare-related tasks and can process both text and images, present an interesting avenue for further investigation. Future work should aim to integrate multimodal data to further enhance diagnostic performance and assess whether models like Med-Palm 2 can address the limitations observed with the current approaches.

There are several limitations when interpreting the results of this study. First, the single-institution nature of the study and the relatively small sample size (*n* = 137) with an uneven distribution of tumor types, particularly the predominance of Glioblastoma cases, may limit the generalizability of the findings across diverse patient populations and reporting practices. Second, the use of structured MRI reports rather than raw imaging data for LLM analysis, while practical for this initial study, represents an artificial constraint compared to real-world diagnostic processes and may not fully capture the nuances available to radiologists interpreting actual images. Third, the study’s design did not assess the LLMs’ ability to adapt to evolving diagnostic criteria over time, nor did it incorporate broader clinical context or patient history into the diagnostic process. Fourth, another limitation of this study is that it did not evaluate the performance of LLMs in differentiating between tumorous and non-tumorous lesions, nor in distinguishing high-grade gliomas from metastases, which are critical challenges for less experienced radiologists. Fifth, this study did not assess the direct image analysis capabilities of LLMs, including newer multimodal models capable of processing both text and images (such as variants of Claude, GPT-4 with Vision, Gemini, and potential future iterations like Llama 3 or 4). All models were provided with structured MRI reports as input. Future research should explore the performance of these advanced multimodal LLMs capable of processing both text and image data directly. Additionally, the absence of formal statistical testing comparing the performance between different LLMs limits the strength of our comparative conclusions; this study primarily relies on descriptive accuracy metrics. Furthermore, this study utilized the LLMs in their default configurations without any task-specific fine-tuning. Fine-tuning models on domain-specific data, such as neuro-oncology reports and guidelines, could potentially enhance diagnostic accuracy, representing an avenue for future research. Lastly, it is important to note that LLMs are rapidly evolving technologies, and the limitations observed in this study may potentially be addressed in the near future as these models continue to advance. These limitations highlight the need for further research with larger, more diverse datasets, multi-institutional collaboration, and the integration of clinical context to evaluate the potential of LLMs more comprehensively the potential of LLMs in assisting with brain tumor diagnosis.

In conclusion, LLMs demonstrate potential as assistive tools in differentiating intra-axial primary brain tumors using structured MRI reports. However, there remains a significant gap between their performance and the diagnostic ability of board-certified radiologists interpreting the actual images. The choice of LLM and the type of tumor substantially impact the results.

## Supplementary information

ELECTRONIC SUPPLEMENTARY MATERIAL

## Abbreviations

API: Application programming interface
CNS: Central nervous system
GPT: Generative pre-trained transformer
IDH: Isocitrate dehydrogenase
LLM: Large language model
MRI: Magnetic resonance imaging
WHO: World Health Organization
