# Supplementary material for: Intra-axial primary brain tumor differentiation: comparing large language models on structured MRI reports vs. radiologists on images
Source: Eur Radiol. 2025 Aug 22;36(2):1594–604. doi: 10.1007/s00330-025-11924-3 (PMC12953250; doi:10.1007/s00330-025-11924-3)
Supplement: Supplementary file 1 — ELECTRONIC SUPPLEMENTARY MATERIAL [file 330_2025_11924_MOESM1_ESM.pdf]

Intra-axial Primary Brain Tumor Differentiation: Comparing Large Language Models on Structured MRI Reports vs. Radiologists on Images

ELECTRONIC SUPPLEMENTARY MATERIAL

"Principal location and size: A 40 mm long mass is observed in the left thalamus-third ventricle (Figure 1). On T2-weighted and FLAIR images, the surrounding high-signal area is not very conspicuous. Heavy T2WI shows an exophytic protrusion from the left thalamus to the third ventricle (Figure 2). The normal pineal gland is seen on the dorsal surface of the mass and is also in contact with the mesencephalic capsule. The mesencephalic cap is compressed caudally (Figure 3). The lateral ventricles are enlarged, and the cerebral sulcus is narrowed, suggesting hydrocephalus. The base of the third ventricle is also deviated caudally. The space between the third ventricle and the midbrain capsule is narrowed (Figure 3). An approach from the anterior third ventricle to the base of the third ventricle is considered possible.

Internal signal: Heterogeneous. Compared to the gray matter, the enhanced area is slightly low-signal on T1-weighted image and slightly high-signal on T2-weighted image (Figure 1), with a high-signal area observed inside the mass on T2-weighted image.

Diffusion-weighted image: The right side of the mass is high-signal, and the ADC value is 0.84 × 10-3 mm2/sec, indicating diffusion limitation (Figure 4).

Hemorrhage and calcification: SWI reveals no low-signal areas suspicious for hemorrhage or calcification. A penetrating structure inside the mass is suggestive of a vein (Figure 5).

Contrast-enhanced T1-weighted image: A somewhat heterogeneous but overall enhancing effect is present on the right side of the mass, which is diffusion-limited (Figure 6). The enhancement effect is not noticeable in the T2-enhanced area on the left side of the mass. There is no abnormal enhancing effect that would raise suspicion of dissemination.

DSC-perfusion (rCBV): The perfusion increase is mild in the enhancement area on the right side of the mass compared to the contralateral normal optic disc. Olea analysis shows that K2 is high (Figure 8), suggesting high permeability in the lesion.

MRS: Elevated Cho, markedly decreased NAA, and elevated Lac are prominent (Figure 9)."
